# Microglia toxicity in preterm brain injury

**DOI:** 10.1016/j.reprotox.2014.04.002

**Published:** 2014-09

**Authors:** Ana A. Baburamani, Veena G. Supramaniam, Henrik Hagberg, Carina Mallard

**Affiliations:** a Perinatal Centre, Department of Physiology and Neurosciences, Sahlgrenska Academy, University of Gothenburg, Sweden; b Centre for the Developing Brain, Department of Perinatal Imaging and Health, Division of Imaging and Biomedical Engineering, Kings College London, St. Thomas’ Hospital, UK

**Keywords:** Perinatal brain injury, Neuroinflammation

## Abstract

Microglia are the resident phagocytic cells of the central nervous system. During brain development they are also imperative for apoptosis of excessive neurons, synaptic pruning, phagocytosis of debris and maintaining brain homeostasis. Brain damage results in a fast and dynamic microglia reaction, which can influence the extent and distribution of subsequent neuronal dysfunction. As a consequence, microglia responses can promote tissue protection and repair following brain injury, or become detrimental for the tissue integrity and functionality. In this review, we will describe microglia responses in the human developing brain in association with injury, with particular focus on the preterm infant. We also explore microglia responses and mechanisms of microglia toxicity in animal models of preterm white matter injury and *in vitro* primary microglia cell culture experiments.

## Highlights

• Microglia responses in the preterm human brain in association with injury.
• Microglia responses in animal models of preterm brain injury.
• Mechanisms of microglia toxicity from *in vitro* primary microglia cell culture experiments.

## 1 Introduction

Microglia are the resident mononucleated phagocytic cells of the central nervous system (CNS) that in their dormant state constantly survey their environment with their extensive processes (see review [1]). Microglia lineage has long been debated, however, recent studies have demonstrated that microglia originate from primitive macrophages in the embryonic yolk sac, prior to hematopoiesis [2]. Upon the formation of embryonic circulation, microglia progenitors enter the neuroepithelium and become established in the brain. Hence, microglia develop independently from hematopoiesis and hematopoietic stem cells [3] and interestingly it has been shown that increased activation/proliferation of microglia is in fact due to local expansion of resident microglial cells as opposed to recruitment of blood monocytes [4], [5]. Although, microglia are largely known as the resident immune cells of the CNS, they are also imperative in normal development of the brain. They are involved in apoptosis of excessive neurons, synaptic pruning, phagocytosis of debris and maintaining brain homeostasis [1], [6], [7].

Hypoxia–ischemia [8], [9] and intra-cerebral administration of excitotoxins such as N-methyl-d-aspartate (NMDA) [10] or ibotenate [11] result in a fast and robust microglia reaction in the developing brain. Neuroinflammation is a dynamic process that plays a key role in the pathogenesis of injury in the developing brain and in response to inflammatory stimuli or tissue injury, microglia secrete a wide range of soluble factors, such as cytokines, substances with excitatory amino acid agonist properties, and glial promoting factors that may influence the extent of subsequent neuronal injury. As a consequence, microglia responses can promote tissue protection and repair following brain injury, or become detrimental for the tissue integrity and functionality. In this review we will describe microglia responses in the human developing brain in association with injury, with particular focus on the preterm infant. We will also consider microglia responses in animal models of preterm white matter injury and the contribution of systemic innate reactions to neuroinflammation. By reviewing *in vitro* primary microglia preparations, we explore mechanisms of microglia toxicity.

## 2 Preterm brain injury

Despite advances in neonatal care there is still significant mortality and morbidity arising from injuries to the developing brain with complications of prematurity [12]. Half of all surviving preterm infants born at or less than 25 gestational weeks, show neurodevelopmental impairment at 30 months of age [13] and at 6 years of age, approximately 40% have cognitive impairment compared to their classroom peers [14]. Magnetic resonance imaging (MRI) studies of infants born preterm have shown that cerebral white matter injury is the predominant pathology of prematurity [15]. However with recent advances in MRI techniques and methodology, it has become clear that white matter injury in the preterm brain is accompanied by abnormal development of the cortical and deep gray matter regions [16], [17], [18], [19]. This complex involvement of gray matter and white matter lesions, which are major determinants of neurologic outcome, is known as “encephalopathy of prematurity”. The periventricular regions are the most common site of preterm white matter injuries recognized on MRI, such as periventricular leukomalacia (PVL), punctate lesions, diffuse excessive high signal intensity, all of which affect white matter development directly, whilst germinal matrix/intraventricular hemorrhage (GMH/IVH) has an indirect affect on the white matter [20]. Injury to the preterm white matter is said to arise from infection/inflammation and hypoxia–ischemia, which could in turn leave premyelinating oligodendrocytes, subplate neurons, late migrating γ-aminobutyric acid (GABA) neurons, and growing axonal trajectories vulnerable to injury [21]. Postmortem studies done over the past decade suggest that activated microglial cells may play a crucial role in mediating injury to the preterm brain [22], [23], [24].

## 3 Microglia in the developing human brain

In the developing human brain, microglial entry into the embryonic forebrain and cerebral cortex is evident as early as 4.5–5.5 gestational weeks through the meninges, choroid plexus and ventricles [25]. Microglial penetration through the vascular component was evident around 10 gestational weeks [26]. The large majority of microglial influx and distribution begins around 16 gestational weeks as ramified cells, and they continue to differentiate and become widely distributed as ramified and active cells up until close to term age [27], [28]. Clusters of transient “resident” populations of amoeboid microglia in the normal preterm brain are prevalent in the periventricular crossroads regions of intersecting callosal, associative and thalamocortical axonal pathways in the white matter [24], [29], and during mid to late gestation the cerebral white matter express high levels of growth associated protein 43, which is associated with active axonal outgrowth [22]. This transient elevation of active “resident” population of microglia in the preterm white matter implies the involvement of microglia in the development and guidance of axonal projections, myelinogenesis and possibly a role in pruning overabundant axons and cells that have failed to reach their developmental destination [27], [29], [30]. It has been suggested that this normal developmental increase in the “resident” population of microglia in the periventricular white matter regions of the preterm brain may be responsible for “priming” this region for inflammatory injury [31].

## 4 Preterm periventricular leukomalacia

One of the first postmortem studies investigating the pathophysiology of preterm PVL demonstrated that injury associated with microglial and astroglial activation is not just contained to the periventricular necrotic foci of the cystic lesion, but is evident as widespread activation in the diffuse component of PVL in the white matter away from the lesion site [32]. Evidence of inflammatory cytokine involvement in preterm white matter injury was reported by Kadhim and colleagues, who showed increased proinflammatory cytokine expression (interleukin (IL)-1β, IL-2 and tumor necrosis factor (TNF)-α) in the white matter of preterm PVL brains [33], [34]. Myelination abnormalities of PVL are believed to be due to arrested maturation of premyelinating oligodendrocytes induced by nitrosative and oxidative mechanisms mediated by microglial cells [32], [35], [36]. There are also neuronal components to the injury, including increase in gliosis and thalamic neuronal loss (60%) together with significant microglial activation [37]. We demonstrated the expression of the innate immune receptor toll-like receptor (TLR) 3 in both glia and neurons in conjunction with preterm white matter injury [38]. A recent postmortem study showed loss of granular neurons in the ventricular/subventricular, periventricular and central white matter regions in preterm PVL [39], which was suggested to be an important contributing factor in neurocognitive deficits seen in preterm brain injury. Further, investigation of the prefrontal cortex in autistic patients showed that there was increased microglia-neuron spatial clustering [40]. However, whether the microglia are involved in neuronal protection and healing or if they are having a deleterious effect on the neurons remains unclear. Nevertheless these finding are of particular interest, as long-term follow up studies of preterm infants have shown that they are at an increased risk of neurocognitive difficulties as well as psychiatric illnesses including autistic spectrum disorder [41], [42].

With advances in neonatal intensive care, there has been a decline in the incidence of classic PVL and non-cystic lesion/diffuse white matter injury is now the predominant type of MRI-defined brain injury in the preterm cohort [43]. Postmortem investigations of diffuse white matter injury show increased microglia activation in both the lesion site and in the deeper white matter regions in this population [44]. Although the diffuse microglial activation was associated with preoligodendrocyte regeneration, these cells were in an arrested state of maturation, similar to that seen in classic PVL, resulting in a reduced pool of mature oligodendrocytes. In the very preterm (26–31 gestational weeks) brain with diffuse injury, the periventricular axonal crossroads region of white matter is characterized by an enlarged microglia population and axonopathy [24]. Hypothetically, the increased microglial activation in the periventricular crossroads region may have a detrimental effect on growing axonal pathways in the white matter during early development.

## 5 Punctate white matter lesion

Whilst preterm punctate white matter lesions are quite common on serial MRI scans (evident in 22% of infants born less than 30 weeks gestational age), the lesions decrease in number by term equivalent age [45]. Although the mortality rate of preterm infants with punctate lesion is low, these infants still show reduced myelination and cortical folding at term. One isolated postmortem case of preterm punctate white matter lesion (identified on postmortem MRI) showed that the lesions corresponded to areas of vascular congestion and infiltration of dense microglial activation [20].

## 6 Preterm germinal matrix/intraventricular heamorrhage

Preterm isolated GMH/IVH with no overt venous parenchymal infarction (as evidenced by postmortem MRI), showed increased microglial activation, cell apoptosis and axonal injury in the periventricular white matter [23]. These results suggest that minor isolated GMH in the preterm brain may still result in deleterious effect on the adjacent white matter through microglial activation. Microglial activation in the periventricular white matter increased with increased severity of hemorrhagic injury, and in addition to increased cell apoptosis and axonal injury, there was evidence of increased TNF-α expression whilst the expression of IL-10 remained unchanged [23], [46]. These results suggest that the persistent activation of microglia in preterm brains with severe GMH/IVH may be a contributing factor to injury through pro-inflammatory mediators.

## 7 Animal models of fetal and neonatal white matter injury

In support of the clinical evidence discussed above, studies in medium- to large-sized animals have frequently demonstrated a link between intrauterine infection/inflammation or fetal asphyxia and microglia activation in the developing brain. Pregnant New Zealand rabbits, on gestation day 28 (term pregnancy: 31–32 days) were injected with lipopolysaccharide (LPS, 20 g/kg) along the length of the uterus between the fetuses. Following maternal LPS exposure, positron emission tomography imaging of the microglia-specific tracer [(11)C]-(R)-PK11195 in one-day old pups demonstrated an increased number of activated microglia, which was associated with the severity of motor deficits in the neonatal rabbit [47]. In midgestation fetal sheep, an age which is similar in brain development to the preterm human, a single intravenous (i.v.) injection of a low dose of LPS (100 ng/kg) resulted in white matter injury and an increase in number of microglia, both in the fetal forebrain and cerebellum [48], [49]. The injury was further characterized by impaired maturation of electroencephalogram and delayed cortical development [50] and a reduction in general systemic metabolism of the fetus [51]. Also following administration of repeated high doses of LPS (1 μg/kg) [52] or low-dose LPS infusion (100 ng, i.v. over 24 h, followed by 250 ng/24 h for 4 days) [53] to fetal sheep there was an increased number of microglia and systemic IL-6 or brain TNF-α-positive cells in the periventricular white matter. Chronic intra-amniotic administration of LPS (for 28 d) caused a moderate to extensive activation/infiltration of microglia/macrophages in the subcortical white matter in six of eight sheep fetuses [54]. Similarly, LPS administered into the uterine artery of late gestation pregnant sheep showed fetal microglial activation and macrophage infiltration. Importantly, no LPS could be detected in the fetus suggesting that neuroinflammation occurred without direct fetal exposure to endotoxin [55]. Repeated neonatal exposure to innate immune mediators [56], or specific cytokines [57], also results in white matter damage in the developing rodent brain when given at an age corresponding to the preterm human infant [58], [59], [60]. Furthermore, non-infectious insults, such as fetal asphyxia, induced by umbilical cord occlusion [48], or cerebral hypoxia–ischemia [61] in midgestation fetal sheep result in marked microglia activation. In neonatal HI, activated microglia are the main producers of pro-inflammatory IL-18, which is activated by caspase-1, which is also expressed by microglia. Indeed, both IL-18 [62] and caspase-1 [63] gene deletion reduces brain injury giving further support to the concept that microglia exert toxic effects under such conditions. Thus, similar to evidence from human post-mortem studies, reactive microglia responses in cerebral white matter and subcortical brain regions are common features in preterm animal models following both infectious and non-infectious insults.

## 8 Combination of systemic inflammation and hypoxia–ischemia

There is considerable evidence that LPS-induced systemic inflammation can exacerbate the neuroinflammatory response and brain injury to cerebral hypoxia–ischemia [64] and excitotoxicity [65]. The LPS effects are dependent on the innate immune receptor TLR-4 [66] and the adaptor protein myeloid differentiation factor 88 (MyD88) [67]. Stimulation of other innate immune receptors also has the capacity to exacerbate hypoxic–ischemic injury. We showed that giving the viral mimic, poly inosinic:poly cytidylic acid (Poly I:C), a synthetic ligand for TLR-3, increased infarct volume and reduced white matter in neonatal mice [68]. Interestingly, enhanced injury was associated with a decrease in reparative M2-like CD11b^+^ microglia, while there was no change in M1-polarized cells. Thus, experimental data propose that triggering innate immune responses systemically may affect both the intensity and characteristics of neuroinflammation. Although the precise underlying mechanisms remain unclear, inhibition of TNF-α [69] and IL-1β [70], the use of anti-nuclear factor kappa-light-chain-enhancer of activated B cells (NF-κB) peptides [71] and immune regulatory peptides [72] alleviate LPS-sensitization of hypoxic–ischemic brain injury, suggesting that inflammatory pathways are important.

## 9 Microglia *in vitro*

Whilst there are some criticism over how comparable *in vitro* preparations of microglia are to the *in vivo* situation [73], [74], *in vitro* studies have allowed for the intimate exploration of key activation and signaling pathways, and the ability to explore the interaction between cell types. Organotypic slice cultures from the hippocampus and cortex have also been utilized to investigate the function of microglia, the benefits of which are better preservation of brain cytoarchitecture, which allows for examining interactions of microglia with neurons and glia following injury [75], [76], [77].

When interpreting findings from *in vitro* studies it is important to consider which type of cell preparation has been used as there are differences in responses between cell lines (such as BV2 and N9, both of murine origin) and primary cultures [78], [79], [80]. It is also important to be aware of the age at which microglia for primary cultures are isolated; protocols range from embryonic (E18), neonatal (postnatal day (P0) to P4), adult (10 weeks) to aged (15 months old), where differences in reactivity, morphology and functionality have been observed [80], [81]. The use of neonatal brains (P0–P4) is by far the most common and Lai et al. [81] found these to be the most reactive in culture in comparison to different ages. It has also been noted that primary microglia cultures obtained from rats are more sensitive to TLR-3/4 stimulation than compared to mice [82]. Numerous agents and pathological conditions have been used to investigate microglial activation and potential for toxicity ranging from bacteria (LPS), cytokines and chemokines (interferon (IFN)-γ, IL-6), proteins, neurotransmitters, reactive oxygen species.

## 10 Activation states of microglia

Microglia are known to have both beneficial and detrimental actions, and in recent years numerous studies have focused on understanding their activation patterns, for detailed reviews see [73], [83], [84], [85]. Briefly, microglial activation states have been sub-classified into classical activation (M1 – tissue defense and pro-inflammatory cytokine production), alternative activation (M2a – tissue repair and anti-inflammatory cytokine production) and acquired deactivation (M2b – immunosuppression). Traditionally LPS has been used to induce robust activation of microglia which leads to the production of TNF-α, inducible nitric oxide (iNOS) and pro-inflammatory cytokines which are all suggested to have cytotoxic downstream effects, characteristic of an M1 phenotype [86], [87]. IL-4 stimulated microglia upregulate genes and proteins that characterize an M2a, reparative phenotype [87].

## 11 Microglial contribution to the pathogenesis of preterm brain injury

### 11.1 Reactive oxygen species and microglia

The immature brain is vulnerable to oxidative stress; and reactive oxygen species (ROS; superoxide (O_2_^−^), hydrogen peroxide (H_2_O_2_)) and reactive nitrogen species (RNS; nitric oxide (NO), peroxynitrite (ONOO^−^)) are produced by and can act to regulate microglia. LPS + IFN-γ stimulated microglia produce NO in a time dependent manner [88]. *In vitro* imaging studies have also shown that in NO producing microglia, O_2_^−^ is a rate-limiting factor in the formation of ONOO^−^ [88]. It has been shown that microglia stimulated with either continuous or bolus H_2_O_2_ results in the production of nitrite, ROS and mitochondrial O_2_^−^. Continuous low H_2_O_2_ also leads to significant production of pro-inflammatory cytokine IL-15, and chemokines (*e.g.* granulocyte colony-stimulating factor, macrophage inflammatory protein-1 and macrophage inflammatory protein 2-alph) [89]. Activated microglia production of intracellular and extracellular ROS is dependent on nicotinamide adenine dinucleotide phosphate (NADPH) oxidase (NOX) [90], [91], [92]. For a detailed review see [93], [94]. Numerous NOX isoforms exist in microglia, but it has been shown that LPS stimulated microglia activate NOX1 and NOX2, which is required for the production of O_2_^−^, NO and iNOS, whilst only NOX1 appears to promote IL-1β production [95]. The activity of NOX1, NOX2 and NOX4 can also be modulated by GABA, glutamate and ATP stimulation, which consequently leads to O_2_^−^ formation, but not iNOS production. Glutamate mediated activation of NOX also contributes to a neurotoxic phenotype in microglia [96].

### 11.2 Excitotoxicity and microglia

Microglia contain purinergic receptors, specifically P1 and P2 receptors, which are activated by adenosine and ATP respectively [81], [96], [97], [98]. Importantly, the P2X_7_ receptor (P2X_7_R; an ionotropic receptor) and ATP binding cassette (ABC) transporters are required for microglial IL-1β production [99]. Haynes et al. [100] found that the metabotropic receptor, P2Y_12_, is required for the fine movement of microglial processes, thereby being important in regulating microglial activation. Extracellular ATP can activate microglia inducing chemotaxis and production of superoxide, nitrate, NOX isoforms, TNF-α and more ATP [81], [96], [101], [102].

Numerous glutamate receptors are present and functional on microglia, including group I, II and III metabotropic glutamate (mGlu) receptors, α-amino-3-hydroxy-5-methyl-4-isoxazolepropionic acid-kainate (AMPA-KA) and NMDA receptors (NMDAR) [103], [104], [105], [106], [107]. For a detailed review about neurotransmitters and microglia see [108]. Upon stimulation of AMPA-KA, mGlu2 and NMDAR microglia are activated and robustly induce TNF-α production [103], [104], [107]. NMDA treatment has also shown to increase production of cellular ROS and NO as well as anti- and pro-inflammatory cytokines [103]. Microglia treated with glutamate or glutamate receptor agonists increase microglial c-fos expression [109]. Stimulation of mGlu3, mGlu5 and group III mGlu does not result in microglial neurotoxicity [104], [105], [106]. Inflammatory activation of microglia (by LPS) results in the release of glutamate, this has been shown to be dependent on lipid peroxidation and NOX but not NO or NOS [110]. Takaki et al. [111] showed that l-glutamate (l-Glu) is released from LPS activated microglia, and when co-cultured with astrocytes, results in decreased uptake of l-Glu by astrocytes leading to significant extracellular l-Glu. Taken together these factors contribute to increased excitotoxicity that can be damaging to neurons and oligodendrocytes.

## 12 Microglial toxicity – effect on oligodendrocytes, neurons and the blood–brain barrier

### 12.1 Microglia and oligodendrocytes

Zajicek et al. [112] investigated the *in vitro* interactions between oligodendrocytes and microglia and found that unstimulated microglia have minimal contact with oligodendrocytes. However, increased contact was observed following microglial stimulation with IFN-γ, or LPS + IFN-γ. Microglial secreted TNF and NO contributed to inducing oligodendrocyte cell death [112], [113].

Miller et al. [114] compared the response of LPS (10 ng/ml) activated microglia in co-culture with oligodendrocyte progenitor cells (OPCs; immature, neural/glial antigen 2 (NG2)^+^ and A_2_B_5_^+^) and oligodendrocytes (mature, galactocerebroside (GalC)^+^ and myelin basic protein (MBP)^+^). LPS activated microglia decreased OPC survival, in contrast resting and activated microglia increased the survival of oligodendrocytes. Domercq et al. [115] found microglia stimulated with a higher dose of LPS (100 ng/ml) inhibited oligodendrocyte glutamate transporters, leading to increased extracellular glutamate and oligodendrocyte (GalC^+^ and O1^+^) death.

Microglia co-cultured with pre-oligodendrocytes (preOL; A2B5^+^, O4^+^) stimulated with LPS leads to increased preOL apoptosis [116], [117]. Li et al. [117] found this was mediated by microglial production of NO and ONOO^−^. PreOLs and preOL-astrocyte co-cultures stimulated with LPS do not result in preOL cell death, highlighting the toxic role of activated microglia. Interestingly, in mixed glial cultures (microglia, preOLs and astrocytes) exposed to LPS, NO is not required for toxicity, rather, in the presence of astrocytes TNF-α production was important for mediating preOL cell death [118].

### 12.2 Microglia and neurons

Neurons have been shown to activate microglia in co-culture [119]. Oxygen glucose deprivation (OGD) stressed cortical neurons activated microglia, which was mediated through extracellular glutamate binding to mGluRII and NF-κB [120], these activated microglia then further elicited neurotoxic effects on neurons, which involved mGluRII, NMDAR, NF-κB and TNF-α [103], [120]. Lai and Todd [121] also found that culture media from mildly injured neurons induced microglial production of IL-1β, TNF-α and NO, this was due to neuronal production of glutamate and ATP. Exposing neuronal cultures to conditioned media from LPS activated microglia induced severe synapse loss, activated caspase-3 activity, DNA fragmentation and neuronal cell death, which was mediated by the MyD88 pathway [87], [122], [123]. Increased neuronal cell death was also seen when neurons were exposed to conditioned media from NMDA treated and mGlu2 stimulated microglia [103], [104]. Studies utilizing microglia-neuron co-cultures have further highlighted the contribution of microglia to neuronotoxicity. Activating microglia with IFN-γ or LPS in co-culture with neurons results in increased neuronal cell death, suggested to be mediated through NO production [124]. ATP stimulated microglia also elicit neurotoxic effects on hypoxic neurons [81].

### 12.3 Microglia and BBB

It has been shown that activated microglia can disrupt and induce injury to constituents of the blood–brain barrier (BBB). Sumi et al. [125] found that co-culturing rat brain endothelial cells with microglia and subsequently stimulating with LPS (10 ng/ml), resulted in fragmented tight junctional immunostaining (zona occludin-1, claudin-5 and occludin), decreased transendothelial resistance and increased sodium-fluorescein permeability, suggesting increased paracellular transport. This was shown to occur *via* NOX mechanisms. Following OGD and reperfusion, the addition of microglia to endothelial cell and astrocyte co-cultures, resulted in increased cell death of endothelial cells. OGD and reperfusion also resulted in increased production of superoxide and H_2_O_2_ [126]. Whether activated microglia influence pericyte morphology or function has yet to be determined.

In summary, there is considerable evidence to suggest that activation of microglia can be neurotoxic and contribute to neuroinflammation seen in the injured preterm brain. However, microglia also mediate critically important functions during normal brain development. To better understand the injurious *versus* protective functions of neuroinflammation, microglia activation states and the possibility of contribution of systemic immune cells in preterm brain pathology need to be determined.

## Conflict of interest

The authors declare that there are no conflicts of interest.

## Transparency document

Transparency document
